# The changing face of percutaneous closure of the patent ductus arteriosus: advances over the last few years

**Published:** 2018

**Authors:** Greyling Adele

**Affiliations:** Division of Paediatric Cardiology, Department of Paediatrics, Dora Nginza Hospital, Port Elizabeth, South Africa

Patent ductus arteriosus (PDA) closure is recommended for patients with moderate-to-large PDAs with significant left-to-right shunting, left-sided volume overload, reversible pulmonary arterial hypertension (PAH) or a previous episode of endocarditis.^1^ In patients with either a small or silent PDA, the decision to close may be less clear. However, closure is still recommended in small, audible PDAs and in selected small, silent PDAs, if so preferred by the clinician and the family, due to the theoretically increased risk for the development of endocarditis.^1^

Since the first transcatheter PDA closure over 50 years ago, it has become the procedure of choice in infants over 5 kg as it is less invasive and more cost effective than surgery and does not leave a surgical scar.^2^ The Amplatzer duct occluders, ADO I and ADO II, are widely used and have been shown to be safe and effective to close moderate-to-large PDAs.^3^ They are probably best suited to Krichenko type A or E PDAs. Similar in design to the ADO I, but with flaring on the pulmonary side of the device, the Occlutech PDA occluder has been proven to be safe and effective, even in the low- to middle-income setting.^4^ With these devices, use is limited in smaller infants due to the size of the delivery system and the large aortic retention disc, which could lead to vascular complications, iatrogenic pulmonary artery stenosis or coarctation of the aorta.

In the ADO II AS device, the occlusive material has been removed, making the device smaller and more flexible. Combined with a lower-profile 4F delivery system, it can be delivered from the aortic or pulmonary side. This makes it particularly well suited for the closure of small- to moderate-sized elongated PDAs (Krichenko type C and D PDAs) in small children. In centres with experienced operators, the outcomes are similar to surgical closure and it is more cost effective.^5^ In smaller PDAs, Cook coils can be used, and although the cost is significantly less, they are associated with increased procedure times and radiation doses, higher risk of migration, residual leak and the need for a redo procedure.^5^

A recent study suggested that a ‘foetal type PDA’, type F, be added to the Krichenko classification and that the Amplatzer vascular plug II (AVP-II) was the most effective device for closure.^6^ They report that PDAs in pre-term infants are similar to those seen during foetal life; typically wide in relation to the descending aortic diameter, and long and tortuous without significant stenosis. Type F PDAs are morphologically most similar to type C PDAs; but they are longer and wider, with a tortuous segment closest to the PA end. The shape resembles a ‘hockey stick’ with an initial mild cranial angulation, followed by a caudal turn at the PA end. They are significantly larger, with the minimal luminal ductal diameter:descending aortic diameter ratio of 0.67 ± 0.12 and device diameter:descending aortic diameter ratio of 1.04 ± 0.18, higher than in any other PDA type.

The biggest change in practice in recent years has been the move to percutaneous closure in smaller and pre-term infants with relatively larger PDAs, even in infants with a weight of less than 2 kg. In centres with experienced operators, outcomes comparable to surgical ligation can be achieved at a lower cost.

In this issue of the journal, Adams and co-workers discuss the development of transcatheter closure of PDAs over a 15-year period at Chris Hani Baragwanath Hospital in Johannesburg (page 246). They found the procedure to be safe and effective in this setting.
